# Factors Influencing Users’ Perceptions of Digital Platform Indispensability: A Comparative Study of Korea and Finland

**DOI:** 10.3390/bs14060502

**Published:** 2024-06-16

**Authors:** Moonkyoung Jang, Shahrokh Nikou, Seongcheol Kim

**Affiliations:** 1 Business School, Gachon University, Seongnam 13120, Republic of Korea; mkjang@gachon.ac.kr; 2 Department Design, Organisation and Strategy (DOS), Delft University of Technology, 2628 CJ Delft, The Netherlands; s.n.nikou@tudelft.nl; 3 School of Media and Communication, Korea University, Seoul 02841, Republic of Korea

**Keywords:** digital platform indispensability, comparative study, platform gap, Korea, Finland

## Abstract

The pervasive integration of digital platforms into daily life has amplified their perceived indispensability. This study investigates the factors influencing this perception across countries with contrasting platform landscapes, focusing on platform quality and usage patterns. We conducted surveys in Finland and Korea, countries representing distinct platform ecosystems. The results revealed higher perceived indispensability in Korea than in Finland, with usefulness and habitual platform use emerging as significant predictors of indispensability in both countries. However, the specific aspects of platform quality influencing this perception diverged. In Finland, the platform’s comprehensiveness and security risk significantly impacted indispensability, while social interaction features played a negligible role. Conversely, in Korea, social interaction features significantly influenced indispensability, while platform comprehensiveness and security risk were non-significant. These findings underscore the multifaceted nature of digital platform indispensability, shaped by the interaction of platform quality and usage patterns. The contextual variations highlighted by our cross-country comparison suggest that a one-size-fits-all approach to platform regulation or user education may be ineffective. Future research should explore these cultural and platform-specific nuances to devise tailored policies.

## 1. Introduction

Digital platforms play a crucial role in connecting people or businesses online, allowing them to engage in activities such as buying and selling goods and services or sharing information [1]. These platforms utilize digital technology to facilitate interactions and are increasingly recognized as essential infrastructure for e-commerce, online communication, and digital social connections [2]. The value of digital platforms cannot be ignored; the role of digital platforms is expanding, connecting and facilitating an ever-widening variety of transactions. With their integrated nature, from e-commerce to fintech, digital platforms diminish boundaries in the digital economy and have become “the operating system of our lives” [3]. Digital platform use, in many ways, is no longer optional; rather, it is an essential tool and the core of the digital ecosystem.

As such, the concept of digital platform indispensability has recently begun to attract significant attention [4]. An indispensable digital platform is one that users find difficult to replace or live without due to its significant impact on their routines, tasks, and interactions. A digital platform can be considered indispensable when it functions as the “operating system” of a user’s life, so that the user perceives the digital platform as essential to their life [5]. 

As digital platforms have become an essential part of people’s day-to-day activities, the gap between local and global platforms has become a significant issue in many countries. The digital platform gap refers to the differences in capabilities, scale, and market reach between the dominant global platforms and the local platforms in each country. This gap poses challenges and creates discrepancies in the digital landscape [6]. For example, the EU faces a platform gap due to the dominance of global platforms like Google in areas such as online search, digital advertising, and mobile operating systems. Lacking its own local digital platform, the EU is addressing this challenge through internal cooperation and active regulation, establishing measures to contend with the impact of global platform dominance in the region [6]. Regulations such as the Digital Markets Act (DMA), Digital Services Act (DSA), and The AI (artificial intelligence) Act constitute efforts to curtail potential abuses of market dominance and ensure fair competition. The DMA was signed into law in 2022 to prevent large platform companies [7]. The Act prohibits companies such as Google, Apple, Facebook, and Amazon, designated as “gatekeepers,” from blocking competitors’ services or monopolizing user data. It also requires platforms to make it possible for consumers to easily switch to other services and includes regulations to create a fair competitive environment. The DMA sets out prohibitions and obligations for large platforms, but it is distinct from traditional competition law and faces uncertain implementation and institutional challenges due to its parallel application with EU competition law [8]. In addition, the DSA was formally passed in 2022 and requires digital service providers to ensure the safety of users and effectively manage illegal content [7]. The act requires online platforms to provide transparency to their users and strengthen procedures for reporting and responding to illegal content. It also strengthens user data protection to minimize possible problems in advertising systems using artificial intelligence. Furthermore, the European Union’s Artificial Intelligence Act was officially proposed by the European Commission in 2021 and formally passed in 2024 [9]. The regulatory proposal created a categorization for the risks of AI systems and set strict requirements for high-risk AI use [10]. For example, technologies that can threaten an individual’s basic rights, such as facial recognition technology, are required to have a higher level of transparency and accountability. In addition, it ensures that AI systems are under human supervision, ensuring that machine decisions are consistent with human values.

In contrast, in China, domestic digital platforms are dominant. This is attributed to government censorship, which has resulted in the development of local platforms [11] such as Baidu for online search, Alibaba for an online commercial marketplace, WeChat for social networking services, and Taobao for consumer goods e-commerce. The dominance of these homegrown platforms in China is a result of unique circumstances, including government policies that have shaped the national digital landscape [6]. The Chinese government had long taken a comprehensive approach to internet regulation, with control measures including the “Great Firewall”—a term for the combination of legislative actions and technologies enforced by the government to regulate the internet in China [11]. This control has allowed China to foster and protect its own tech industry, creating an ecosystem where local companies could grow and adapt to the specific needs and regulations of the local market without direct competition from foreign companies [11]. This situation reflects broader themes of digital sovereignty, where governments seek to maintain control over the data and digital services used by their citizens. It raises important questions about the balance between controlling information flows for national security and cultural preservation versus the benefits of a free and open internet [12].

Other countries are witnessing platform battles where local and global platforms compete. South Korea (hereafter referred to as Korea) serves as a notable example [13]. Naver currently maintains a leading position with a market share of 57.7% in the search engine market, but it is encountering challenges to its dominance as Google gains traction. This dynamic reflects the competitive landscape as local and global platforms vie for supremacy in the national market [14]. Similarly, digital platforms have become an integral and indispensable part of Finland’s urban development and the emergence of national innovation hubs [15]. 

While the impact of various factors on the indispensability of digital platforms may differ based on the country’s information and communication technology (ICT) environment, there is a notable absence of cross-country comparisons. There is a clear need for research that systematically compares the role and significance of digital platforms across different countries, considering the varying influences of factors in different ICT landscapes.

This study examined the factors influencing users’ perceived indispensability of digital platforms and investigated potential variations in this perceived indispensability of users in countries with distinct digital platform gaps. We selected Korea and Finland as the focus of our comparative analysis due to their contrasting digital platform ecosystems and their status as technological leaders within their regions. Korea, with its dominant local platforms like Naver and Kakao, provides a unique context to explore the role of domestic digital services in everyday life. In contrast, Finland, influenced predominantly by global platforms such as Google and Facebook, offers insights into the impact of global digital services within the EU regulatory framework. This selection allows for a comprehensive examination of how different market dynamics and cultural contexts influence the perceived indispensability of digital platforms. 

Aligning with Hoffman et al.’s 2004 study [5], these factors were categorized into two layers: the characteristics of the digital platform (such as comprehensiveness, usefulness, and security risk) and the degree of use of the digital platform (including usage habits and daily frequency). Lastly, this study explored how the impact of these factors on the indispensability of digital platforms differed between countries with varying digital platform gaps. Finland, representing the EU, and Korea were chosen as case studies due to their differing digital platform landscapes. Therefore, our research questions are as follows:

**RQ1:** How does the perceived indispensability of digital platforms vary between Korea and Finland?
**RQ2:** What factors determine how indispensable digital platforms are considered in Korea and Finland?
**RQ3:** To what extent do the relative influences of specific factors on platform indispensability differ between Korea and Finland?

To understand the differences in how digital platforms are valued across cultures, we conducted surveys in both Finland and Korea. We then compared the average responses using a *t*-test to pinpoint any significant gaps. Further, we employed hierarchical regression analysis to explore the specific factors influencing these perceptions in each country.

Focusing on the growing disparity between global tech giants and local platforms, this paper first provides an overview of the indispensability of digital platforms. Next, the research hypotheses are introduced, and the research model is presented. Section 3 presents research data and variables, while Section 4 offers descriptive and conceptual results. Section 5 comprises the discussion and conclusion, along with the presentation of limitations and future research directions.

## 2. Literature Review and Research Hypotheses

### 2.1. Digital Platform Indispensability

In the context of this study, digital platform indispensability refers to the degree to which users perceive a digital platform as essential to their daily lives. An indispensable digital platform is one that users find difficult to replace or live without due to its significant impact on their routines, tasks, and interactions. The concept of indispensability is a defining trait for items that play a pivotal role in shaping individuals’ lives [16]. Research on the indispensability of information and communication technologies (ICT) explores the diverse ways these technologies become embedded in and essential to daily life, examining factors like user motivations, usage patterns, and impacts on perception and behavior [5,16,17,18,19,20]. The seamless integration of advanced ICT into daily life has rendered various information technologies indispensable [21]. First, the internet, a representative ICT, drove the internet revolution in the late 1990s, becoming an indispensable aspect of daily life, facilitating work, shopping, entertainment, and social networking [5]. Research on the indispensability of the internet involves understanding user purposes, perceptions, frequency of use, and utilization methods [5,22]. Beyond the internet, studies have explored the indispensability of other ICTs such as mobile phones and television. Experiments with cell phones, the internet, and television have shown that the sense of indispensability for each technology varies and affects people’s perceptions of its use and absence [16]. The concept of “smartphone essentiality” has also been studied, with research examining over-reliance on smartphones and comparing differences in smartphone indispensability across countries in relation to national happiness levels [23]. In addition, research has examined the indispensability of mobile media, as it is increasingly important in the modern world, especially for urban dwellers and the privileged [21]. 

As digital platforms become more integrated into our daily routines, there has been a growing research focus on the concept of digital platform indispensability, which examines the extent to which a platform assumes a vital and irreplaceable role in users’ lives, granting access to crucial functionalities and essential services that are perceived as challenging to replace [24]. This surge in research, exploring diverse aspects like the nature of user interactions, platform functionalities, and their dynamic evolution, is evident across a range of countries. 

One area of indispensability research regarding digital platforms has examined their essential role in communication between people. Jansson (2015) [25] explores the various sociocultural factors that influence the perception of different digital platforms—email, video calls, online chat, and social media—as essential elements of social technology. Drawing on insights from a nationwide survey in Sweden, the study shows that email and video calling have unique cultural characteristics for communication, as opposed to online chat or Facebook. It also highlights that the preference for email is significantly higher among people with higher levels of education and a more globalized lifestyle.

Scholars are also exploring other dimensions of digital platform indispensability, examining their diverse functionalities in areas like commerce, education, entertainment, and even public services [26]. For example, WeChat in China was initially designed as a messenger app but has evolved into an indispensable tool due to its dual nature as a digital platform and infrastructure, now offering a vast array of services beyond messaging such as mobile payments, transportation booking, and online shopping, further emphasizing the context-specific factors that contribute to platform indispensability [27]. This comprehensive range of functions, providing access to critical services and seamlessly embedded in daily routines, has contributed to WeChat’s widespread adoption, making it an integral part of many people’s daily lives in China and exemplifying the growing influence of multifunctional platforms in shaping user dependence. Another example is TikTok, a popular social media platform that originally became famous for allowing users to create and share short-form videos [12]. TikTok has expanded its scope and utility beyond short-video content, becoming increasingly indispensable by incorporating diverse services including e-commerce, online education, marketing, and tourism services.

### 2.2. Digital Platform Gaps between Local and Global Platforms

The degree of indispensability associated with a digital platform may be positively correlated with the existence of a “digital platform gap”—a disparity in capability, size, or market share between local and global platforms within a specific region or country. Highly indispensable platforms are likely to occupy a dominant position within their region [27], potentially exacerbating the existing gap as their indispensability continues to grow. This occurs because global platforms leverage their existing dominance to further entrench themselves, while local platforms often struggle to maintain competitiveness. Understanding the relationship between platform indispensability and the local-global gap provides valuable insight into the acceptance and development trajectories of digital platforms within specific regions. Ultimately, these dynamics influence the nature of competition and cooperation within the digital ecosystem.

The nature of a country’s response to a highly indispensable digital platform often hinges on its origin, whether global or local [28,29]. This complex dynamic stems from the recognition that digital platform development transcends mere technological advancement; instead, it emerges from a nuanced interplay between government policies, specific national historical contexts, and the strategic maneuvers of platform companies [13]. Consequently, a granular understanding of a nation’s unique societal and regulatory landscape is indispensable for analyzing the specific facets of platform indispensability within that context [27]. In countries where a single platform enjoys significant indispensability, concerns naturally arise regarding potential excessive public dependence. This stems from the inherent correlation between high platform indispensability and diminished digital ecosystem competition, ultimately leading to enhanced platform influence.

Greater indispensability associated with global digital platforms, as compared to their local counterparts, often ignites heightened concerns regarding burgeoning public dependence in various countries. This phenomenon is exemplified by Google’s perceived indispensability in numerous countries beyond the United States, prompting multifaceted discourse surrounding the potential need for, and effectiveness of, regulatory measures [30,31,32,33,34]. The European Union (EU) offers a prominent case study, actively scrutinizing Google’s practices in data collection, utilization, and user rights protection within its own jurisdiction, where the platform is dominant. Notably, in 2018, Google faced accusations of anti-competitive tactics involving pre-installation mandates for its search app and Chrome browser on the Android operating system, alongside stifling competition for developers of alternative search engines and browsers. Consequently, the EU Commission levied a €4.3 billion fine, deeming Google’s actions an abuse of market dominance. Furthermore, Google was compelled to comply with stipulations dictating the provision of alternate search engine and browser options to Android users. The EU continues to investigate allegations of Google’s abuse of its market dominance within the digital advertising sphere, focusing on claims of preferential treatment for its own advertising services and discriminatory practices against competitors. Should these accusations be substantiated, Google could face additional financial penalties and potentially market-opening mandates.

In regions characterized by high indispensability of local digital platforms, diverse perspectives emerge regarding regulatory approaches. Proponents of national support argue that fostering domestic platform indispensability can enhance regional competitiveness and bridge the gap with global competitors [27]. This perspective aligns with the leveraging of indigenous platforms as instruments for technological autonomy and sovereignty. However, concerns regarding the potential pitfalls of local monopolies also fuel opposing calls for robust antitrust measures [27]. China exemplifies this complex dynamic, oscillating between stringent control over platforms such as WeChat, aimed at securing domestic dominance, and aspirations to establish WeChat as a pillar of global digital infrastructure, creating a tension between national autonomy and international outreach [35]. Notably, the Chinese government’s contrasting approach to TikTok, characterized by active involvement in content moderation and data regulation, suggests a potential shift towards more nuanced control mechanisms [12].

Certain regions exhibit a convergence in the indispensability of both global and local digital platforms. In Korea, Naver has established itself as a ubiquitous platform, offering a range of services such as search, news, e-commerce, and navigation. However, Google’s influence has witnessed a gradual ascent in recent years. Leveraging its market dominance in domains such as the Android operating system, search, and advertising, Google’s footprint within the Korean digital landscape is expanding, potentially leading to its own classification as an indispensable platform. This rising indispensability of Google raises concerns regarding its potential impact on the dynamics of Korea’s digital market and consumer choice. Should Google exploit its market power to stifle competition or engage in discriminatory practices, it could impede the healthy development of the digital ecosystem and infringe upon consumer rights and interests. Recognizing this possibility, the Korean government is actively exploring regulatory measures to curb Google’s market dominance, while Naver is concurrently undertaking strategic initiatives to maintain its competitive edge.

In Korea, local platforms like Naver dominate some digital platform domains, while in Europe, including Finland, major digital services are predominantly run by large U.S. companies such as Google, Facebook, and Amazon [6]. The experience of Nokia offers insight into why Finnish local platforms may struggle to achieve similar dominance. Nokia’s decline in market competitiveness can be attributed to several factors: leadership transitions that delayed the embrace of new technologies and market trends, an organizational culture that hindered rapid innovation, and a broad product range that scattered its strategic focus [36]. Additionally, Nokia’s delay in creating service-based systems and its struggles to adjust to global market conditions, especially in the U.S., offer a cautionary case study [36]. These points emphasize the need for agile management, focused innovation, and strategic ecosystem development to compete with global tech giants.

Consequently, the indispensability of a digital platform emerges as a pivotal concept, intertwined with the dynamics of competition and cooperation within the digital ecosystem while simultaneously implicating the protection of user rights and interests [1,13]. This complex construct transcends the sole domain of a platform’s technical prowess, extending to encompass the nuanced realm of user perceptions. Notably, such perceptions are likely to exhibit variance across national contexts, shaped by a confluence of factors including cultural backgrounds, societal structures, and prevailing economic conditions [37,38], further compounded by the existence of digital platform gaps [27]. Therefore, comparative analyses across countries offer a valuable avenue for deconstructing the multifaceted influences that shape user perceptions and their subsequent impact on the indispensability of digital platforms.

### 2.3. Research Hypotheses

#### 2.3.1. The Quality of the Digital Platform

Digital platforms are progressively becoming essential for all aspects of daily life, encompassing work, transportation, leisure, and communication, while continuously offering an increasing variety of functions. Thus, the quality of the digital platform itself is a key factor in it becoming an indispensable part of people’s lives [39]. The quality of the digital platform can be measured in various aspects. First, the quality of the digital platform itself can be considered in terms of functionality, or the provision of various functions that meet user needs [25]. The more functions a digital platform provides, the more people will use it in more contexts [40,41]. For example, a digital platform that provides only business functions will be used only when people are working; a digital platform that provides only entertainment functions will be used only in their leisure time. However, a digital platform that provides both work and entertainment functions, while likely more complex, will be used in both contexts. Therefore, the more functions are available on a single digital platform, the more indispensable people think of the digital platform. Thus, we formulate the following research hypothesis.

**H1.**  *The perceived comprehensiveness of a digital platform positively influences its perceived indispensability.*

In addition, the quality of a digital platform can be considered in terms of its usefulness [42,43], in other words, the effectiveness of the digital platform’s functions. The higher the usefulness of a function is, the greater the value the digital platform can provide to users [44]. The more a digital platform is perceived as useful, the more it becomes indispensable [45]. Therefore, the following research hypothesis was established.

**H2.**  *The perceived usefulness of a digital platform positively influences its perceived indispensability.*

Next, the quality of the digital platform can be examined in terms of the level of social interaction through the digital platform [4,25]. The primary function of digital platforms lies in facilitating interactions between people, and the network effect of the digital platform can be thought of as the most important capability to achieve an absolute competitive advantage [46]. The most important requirement for achieving network effect is the number of users [39]; when the number of users exceeds a certain critical mass, new entrants are forced to use the digital platform, and existing users are forced to continue using the platform because the cost of moving to other digital platforms increases [42]. The network effect of a digital platform is achieved through social interaction [4]. The more users there are, the more opportunities for social interaction there are, which, in turn, forms a virtuous cycle that induces user participation [47]. Therefore, the more people perceive that social interaction on a digital platform is high, the more inevitably they perceive the indispensability of the digital platform. Accordingly, the following research hypothesis was formulated.

**H3.**  *The perceived social interaction of a digital platform positively influences its perceived indispensability.*

Digital platform quality can also be considered in terms of security [42,48]. The use of digital platforms inevitably results in the accumulation of individual’s personal and behavioral information within the platform’s logs [49]. Therefore, digital platform indispensability is affected by the user’s perception of how digital platform owners are handling data security, including leak prevention and data ownership issues related to data provision to third parties [50]. The safer and more stable a digital platform’s operation is, the more the user will trust it [51]. In other words, the higher the security risk is, the more anxious people will be about using the digital platform, which will soon negatively affect digital indispensability. Thus, the following research hypothesis was formulated.

**H4.**  *A digital platform’s perceived security risk negatively influences its perceived indispensability.*

#### 2.3.2. Digital Platform Usage

In addition to the functional aspects of digital platforms, usage patterns constitute a significant determinant of their perceived indispensability [5]. Frequent use signifies regular, need-driven engagement, which integrates the platform into users’ daily lives [52]. For instance, frequent multi-purpose utilization—for work (e.g., email), communication (e.g., social media), or leisure (e.g., streaming platforms)—elevates the platform’s significance and increases platform dependence [53,54]. Inability to access email might hinder work progress, and absence from a social media platform could impede social interactions. Therefore, the more frequently a digital platform is used, the more people will perceive it as indispensable. Thus, we posited the following research hypothesis.

**H5.**  *The use frequency of a digital platform positively influences its perceived indispensability.*

In addition, habitual use of digital platforms affects their indispensability [52,55]. Habitual use of a digital platform means that it is not only used on an as-needed basis but as a natural part of daily life. Habitual users perceive the platform as an intrinsic element of their life, deeply embedded in their daily routine; for example, if it has become a habit to use a digital platform before going to bed or upon waking. In such cases, the platform becomes essential, its absence potentially causing discomfort or disrupting the user’s established routine. Therefore, habitual use of a digital platform can influence people to perceive it as indispensable. Therefore, the following research hypothesis was established.

**H6.**  *Habitual use of a digital platform positively influences its perceived indispensability.*

**Figure 1:**
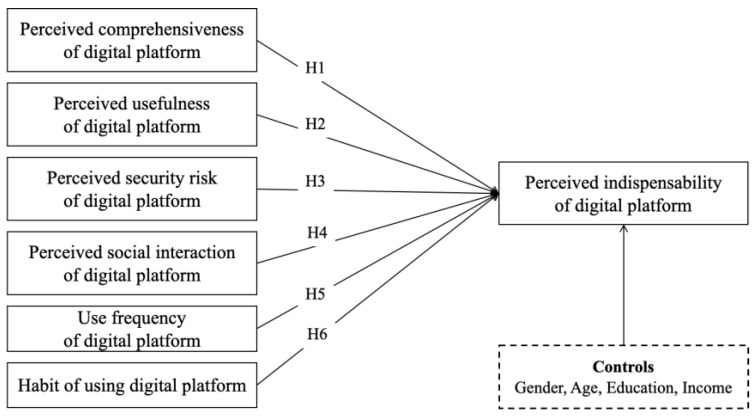
Conceptual research model.

Based on the above theoretical discussions and the hypotheses, our research model is shown in Figure 1.

## 3. Methodology

### 3.1. Data

#### 3.1.1. Sampling Method

Finland and Korea were selected for our international comparative study because they are leading ICT powerhouses in Asia and Europe, respectively, and have been used for cross-country comparisons in previous studies [56,57]. These two relatively small countries also have domestic brands that have managed to compete in the global ICT ecosystem, which is mainly led by companies from the United States and China. Both countries are home to global ICT manufacturers such as Samsung Electronics and Nokia, as well as promising tech startups. Given their world-class ICT infrastructure, both countries also lead the world in internet usage and smartphone penetration rates [58]. Although they share similarities, the two countries exhibit an apparent difference in digital platform gaps. Within the EU, Finland confronts a platform gap arising from the dominance of global platforms like Google in key areas such as online search, digital advertising, and mobile operating systems. Without a robust local counterpart, Finland seeks to mitigate the impact of international companies’ regional platform dominance [6]. Korea offers a relevant, contrasting case. The domestic platform Naver reigns supreme in the search engine market; however, its lead is under increasing pressure from Google’s burgeoning footprint. This dynamic exemplifies a fiercely competitive landscape where local and global platforms grapple for primacy [14].

#### 3.1.2. Survey Instrument and Validation

To answer our research questions, we conducted an online survey in both countries using two identical online survey questionnaires. In Finland, the survey was administered in English, and the data were collected between May and July 2023 through a multifaceted approach, utilizing university notice boards, student mailing lists, and the authors’ established social media networks. For the Korean sample, the survey was administered in Korean, and data collection occurred in November 2022, in collaboration with professional research agency Micromill Embrain, using a stratified random sampling method to ensure the representativeness of the sample. The total sample included 153 responses from Finland and 214 responses from Korea. 

The study adopted a three-part questionnaire design. The first section solicited demographic information relevant to the analysis, including gender, age, highest level of education, and average monthly household income. These variables served as control variables in the subsequent modeling. The second part focused on platform usage, with participants reporting on their daily frequency of engagement with the dominant platform in their respective countries (Google in Finland and Naver in Korea). Respondents reported their daily use frequency of the digital platform on a 5-point Likert scale, ranging from 1 “less than 10 times” to 5 “more than 40 times”. 

Building upon prior studies, the final section of the questionnaire used adapted survey items to capture respondents’ perceptions of platform quality within the context of our research model. Perceived indispensability is defined as the extent to which users perceive a digital platform as essential or crucial to their daily lives, making it difficult to replace or live without [16,59]. Perceived comprehensiveness is defined as the extent to which a digital platform is perceived as comprehensive, offering a wide range of functions and services [60,61,62]. Perceived usefulness is defined as the degree to which a user believes that using a particular digital platform enhances their performance and efficiency in daily activities [63,64,65]. Perceived security risk is defined as the degree to which users perceive potential security risks and privacy concerns associated with using a digital platform [48,63,64,65]. Perceived social interaction is defined as the extent to which a digital platform facilitates social interactions, communication, and community building among its users [66,67]. Habitual use is defined as the extent to which the use of a digital platform has become a routine or habitual part of a user’s daily life [55,61,65,68,69].

The total number of survey items in the last part was 21: 5 items on perceived indispensability of the digital platform adopted from [16,59]; 4 items on perceived comprehensiveness of the digital platform adopted from [60,61,62]; 4 items on perceived usefulness of the digital platform adopted from [63,64,65]; 3 items on perceived security risk of the digital platform from [48,63,64,65]; 5 items on perceived social interaction of the digital platform from [66,67]; and 5 items for habitual use of the digital platform from [55,61,65,68,69]. Respondents indicated agreement with statements on a 5-point Likert scale, from 1 “strongly disagree” to 5 “strongly agree”.

#### 3.1.3. Ensuring Data Reliability and Validity

Prior to conducting the main analysis, composite reliability (CR) and average variation extracted (AVE) were measured for all respondents and Finnish/Korean respondents to verify the convergent and discriminant validity of variable measurement. The composite reliability level of each variable was 0.7 or more, and the average variance extracted value was 0.5 or more, indicating satisfactory reliability [70]. Additionally, a pre-test of the survey instrument was conducted with a small sample (30 participants) in both countries to ensure clarity and relevance of the survey items. Feedback from the pre-test was used to refine the questionnaire further.

### 3.2. Descriptive Analysis

We obtained 153 usable responses from Finland and 214 from Korea. Table 1 presents the demographic information of all respondents in each country. The Finnish sample consisted of 80 (52%) female respondents, 68 (44%) male respondents, and 5 who identified as “other”. The Korean sample contained 142 (66%) female respondents and 72 (34%) male respondents. Most of the respondents were within the age range of 20–40 years (84% for Finnish, 60% for Korean), with an average age of 36.44 for Finnish respondents and 39.63 for Korean respondents. Most respondents had a bachelor’s degree or higher (77% for Finnish, 63% for Korean). Many respondents reported monthly household income between USD 1500 and USD 4500 (69% for Finnish, 58% for Korean).

Table 2 presents the descriptive statistics of all responses regarding all the constructs presented in Figure 1. The indispensability of the digital platforms was rated 3.72 on average; the average comprehensiveness of the digital platforms was rated 3.99; the perceived usefulness of the digital platforms was rated highest with an average value of 4.03; the perceived security risk of the digital platform was rated 2.74 on average; the perceived social interaction of the digital platform was rated 3.62 on average; and the habitual use of the digital platform was rated 3.95 on average. The average daily use frequency of the digital platform was 2.61 on a scale ranging from 2 “10—less than 20 times” to 3 “20—less than 30 times”.

The correlation analysis results are presented in Table 3. The perceived indispensability of the digital platform was not significantly related to any demographic information. The perceived indispensability of the digital platform was positively related to the perceived comprehensiveness of the digital platform (correlation = 0.359; *p* < 0.001), the perceived usefulness of the digital platform (correlation = 0.590; *p* < 0.001), the perceived social interaction of the digital platform (correlation = 0.436; *p* < 0.001), the habitual use of the digital platform (correlation = 0.646; *p* < 0.001), and the daily use frequency of the digital platform (correlation = 0.319; *p* < 0.001). Additionally, the perceived indispensability of the digital platform was negatively related to the perceived security risk of the digital platform (correlation = −0.155; *p* < 0.05).

## 4. Results

To address our research questions and test the hypotheses, we conducted a twofold analysis: two-sample *t*-test and hierarchical regression. In detail, first, to answer the RQ1, we conducted an independent two-sample *t*-test to analyze the differences in the perceived indispensability of the digital platforms and suggested variables. Second, to answer the RQ2 and test the hypotheses, we conducted hierarchical regression to test our research model and investigate whether the suggested variables affected the perceived indispensability of the digital platforms in Finland and Korea. Lastly, to answer RQ3, we compared the coefficients of the two countries.

### 4.1. Difference between the Mean of the Two Countries (RQ1)

The *t*-test results comparing the responses from Finland and Korea are presented in Table 4. The indispensability of the digital platform was significantly higher in the Finnish responses (mean = 3.87) compared to Korean responses (mean = 3.62). This result indicates Finns recognize the dominant digital platform as more indispensable in their lives than do Koreans. Regarding platform quality, the comprehensiveness and security risk of the digital platform did not differ significantly between Finns and Koreans. The social interaction and perceived usefulness of the digital platform were significantly higher in Finnish responses (mean of Social interaction = 3.70; mean of Perceived usefulness = 4.10) compared to Korean responses (mean of Social interaction = 3.57; mean of Perceived usefulness = 3.98). These results suggest that, compared to Koreans, Finns are more aware of the social interaction function of digital platforms in their daily lives and more aware of the usefulness of digital platforms. In terms of platform usage, the habitual use and daily use frequency of the digital platform were significantly higher in Finnish responses (mean of Habit = 4.04; mean of Daily use frequency = 2.51) compared to Korean responses (mean of Habit = 3.89; mean of Daily use frequency = 1.91). These results indicate that compared to Koreans, Finns recognize that they use digital platforms more habitually and more frequently in their daily lives. The item with the greatest difference between the mean of the two countries among the surveyed items was daily use frequency of the digital platform, followed by the indispensability of the digital platform.

### 4.2. Analysis Results (RQ2 & 3)

To evaluate the hypotheses, hierarchical regression analyses were performed utilizing Stata 14. This method facilitated the examination of the incremental variance in perceived platform indispensability accounted for by various predictor sets, systematically arranged according to theoretical rationale and the specific aims of the research. 

While Structural Equation Modeling (SEM) offers considerable advantages for examining complex relationships and latent variables, it is particularly geared towards scenarios that require the evaluation of latent variable reliability and adjustments for measurement errors [71,72]. SEM is ideal for research models that involve intricate interrelationships among variables. SEM is ideal for research models that involve intricate interrelationships among variables and latent constructs. 

In contrast, our study focused primarily on exploring the direct causal relationships between variables. Therefore, multiple regression analysis (MR) was deemed more suitable for our research objectives. Hierarchical regression, a variant of multiple regression, was specifically chosen due to its ability to sequentially introduce variables into the regression equation. This method allows for an ordered exploration of the data, aligning with our research objectives to prioritize causal relationships and eliminate potential confounding influences. Hierarchical regression analysis provides several key advantages. First, hierarchical regression allows for the systematic examination of the incremental variance explained by different sets of predictors [73]. This approach enables us to understand how each block of variables contributes to the overall model, providing a clear and structured analysis of the predictors’ impact. Second, the hierarchical approach aligns well with our theoretical rationale, allowing us to introduce variables in a sequence that reflects our research aims. This method helps in isolating the effects of each predictor set and enhances the clarity and relevance of the findings. Third, our primary focus was on direct causal relationships between observed variables, making hierarchical regression an appropriate choice [74]. This method efficiently handles the examination of direct effects without the need for latent variable modeling. Lastly, hierarchical regression provides clear partitions of the total variance of the outcome variable, indicating the proportion of variance accounted for by each predictor. This clarity was crucial for interpreting the results in line with our research goals.

Based on the hypotheses, three models were constructed for analysis. The first model, serving as the baseline, incorporates individual characteristics such as gender, age, education, and income as control variables. This setup allows for an initial understanding of demographic factors’ influence on the outcome variable. Progressing to the second model, core variables pertinent to the platform’s quality—comprehensiveness, usefulness, security risk, and social interaction—are added. This inclusion is designed to examine the direct effects of platform attributes on its perceived indispensability beyond the demographic controls. The third and final model integrates two additional predictors, habitual usage and usage frequency, to capture the impact of user engagement on their perceptions of indispensability.

The analysis of the Finnish responses is presented in Table 5. Model 1 in Finland showed 2.7% explanatory power. The results of Model 1 indicated that the effects of individual characteristics on the indispensability of digital platforms were not significant. Model 2 applied to the Finnish sample showed 28.1% explanatory power. The results indicated that the effects of individual characteristics on the indispensability of digital platforms were not significant. Perceived usefulness had a significantly positive impact on the perceived indispensability of the digital platform and perceived security risk had a significantly negative impact. Comprehensiveness and social interaction did not significantly affect the perceived indispensability of the digital platform. Model 3 applied to the Finnish sample showed 36.2% explanatory power. Regarding the demographic control variables, the results again indicated that the effects of individual characteristics on the indispensability of digital platforms were not significant. Regarding platform quality, the results indicated that comprehensiveness and security risk both had a significantly negative impact on the perceived indispensability of the digital platform, while the effect of usefulness was significantly positive. Regarding platform usage, only habitual use had a significantly positive impact on the indispensability of the digital platform. The results across the models demonstrate a significant increase in R² and adjusted R² values, indicating that each subsequent model, with its respective set of variables, significantly enhanced the explanatory power of the analysis. The ΔF statistics further confirmed that the additions at each step substantially improved the model fit, reinforcing the hierarchical regression’s utility in elucidating the layered contributions of various predictors to the dependent variable’s variance.

Results of the analysis of the Korean responses are presented in Table 6. Model 1 for the Korean sample showed 2.9% explanatory power. Here, too, the effects of the control variables (i.e., individual characteristics) on the indispensability of digital platforms were not significant. Model 2 for the Korean sample showed 45.2% explanatory power. Again, the effects of individual characteristics on the indispensability of digital platforms were not significant. Regarding platform quality, the results indicated that perceived usefulness and social interaction had a significantly positive impact on the perceived indispensability of the digital platform. The effects of comprehensiveness and security risk did not significantly affect perceived indispensability. Model 3 for the Korean sample showed 59.2% explanatory power. Here, too, the effects of individual characteristics on the indispensability of digital platforms were not significant. Among the platform quality factors, usefulness and social interaction had a significantly positive impacts on the indispensability of the digital platform. The results indicated that habitual use was the only platform usage factor that had a significantly positive impact on the perceived indispensability of the digital platform. Consistent with the findings from Finland, the results from Korea across the models also showed a significant enhancement in both R² and adjusted R² values, in addition to notable increases in the ΔF statistics.

**Figure 2:**
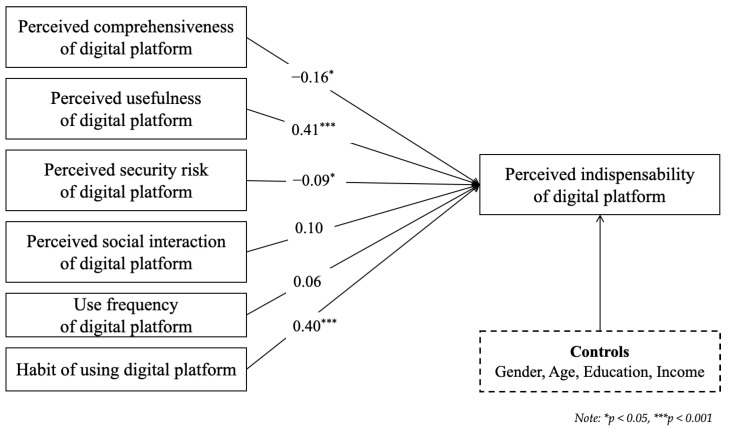
Regression coefficients for Finland.

**Figure 3:**
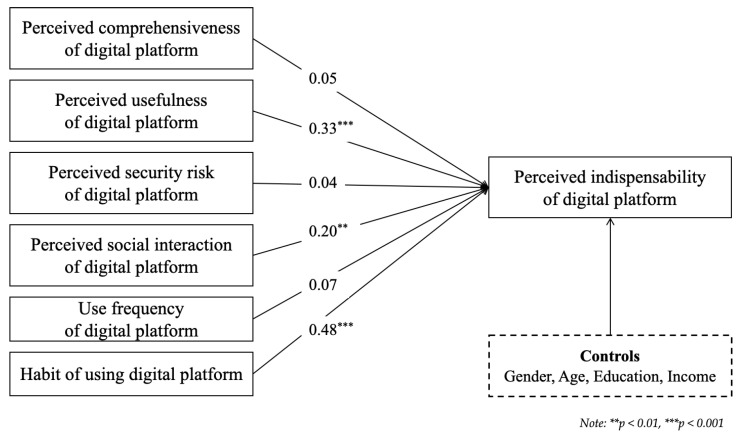
Regression coefficients for Korea.

To identify the effect differences between Finland and Korea, Table 7 presents the results of Model 3 for the two countries. There were no significant effects of individual characteristics on the indispensability of digital platforms in either country; however, there were differences in the effect of platform quality. For Finland, while usefulness had a positive effect, comprehensiveness and security risk had a negative effect. Meanwhile, for Korea, usefulness and social interaction had positive effects, but the effects of comprehensiveness and security risk were not significant. Regarding platform usage, habitual use had a significantly positive impact on the indispensability of the digital platforms in both countries, while the effect of daily usage frequency was not significant in either. Figure 2 and Figure 3 visually represent the significant regression coefficients from the hierarchical regression analysis for Finland and Korea, respectively.

### 4.3. Discussion

Our study aimed to investigate the factors influencing the perceived indispensability of digital platforms in Korea and Finland. The hierarchical regression analysis revealed notable differences between the two countries, highlighting the unique cultural and technological contexts that shape users’ perceptions.

In Finland, the results indicated that perceived usefulness and habitual use significantly influenced the indispensability of digital platforms, while comprehensiveness and security risk had a significant negative impact. These findings align with previous studies that emphasize the importance of functional benefits and security concerns in technology adoption [4,44]. The negative impact of comprehensiveness may indicate that overly complex platforms can overwhelm users, leading to reduced perceived indispensability. This finding aligns with studies that highlight the potential drawbacks of excessive functionality [75]. The strong positive impact of usefulness suggests that Finnish users prioritize practical and efficient features that enhance their daily activities. This finding is consistent with the Technology Acceptance Model (TAM), which posits that perceived usefulness is a critical determinant of technology adoption [43]. Security concerns significantly negatively impacted indispensability, reflecting the high importance Finnish users place on privacy and data protection. This is consistent with findings from [76], which emphasize the need for robust security measures to gain user trust. The significance of habitual use underscores the role of routine and automatic behaviors in establishing platform indispensability. This is supported by the Habitual Use Theory, which suggests that repetitive use can lead to a sense of indispensability [52,68].

In Korea, perceived usefulness and social interaction were significant positive predictors of indispensability, while comprehensiveness and security risk were not significant. These results reflect the unique cultural context of Korea, where social connectivity and communication play a crucial role in digital platform use [77]. Like Finland, usefulness was a significant factor, highlighting the universal importance of functional benefits. This finding supports the broader applicability of the TAM model [43]. The significant positive impact of social interaction in Korea underscores the importance of platforms as tools for social engagement and community building. This is consistent with research that emphasizes the role of social features in enhancing user experience and satisfaction in collectivist cultures [37]. The non-significance of comprehensiveness and security risk may reflect a higher tolerance for platform complexity and lower sensitivity to security issues among Korean users. This could be due to the dominant presence of local platforms like Naver, which are perceived as more trustworthy [78].

These findings offer several important implications. First, this research reveals that the perception of the indispensability of digital platforms may exhibit variations across countries. In this study, the perception of the indispensability of digital platforms in Finland surpassed that of Korea. This disparity can be ascribed to factors such as the pronounced dominance of global platforms in Finland. These global platforms play a pivotal role in Finnish society, influencing aspects of daily life such as social interaction, usefulness, habitual usage, and daily usage frequency, all more so than in Korea.

Second, this research supports the results of previous studies on the indispensability of digital platforms. The indispensability of digital platforms is influenced by various factors, including functional aspects, such as communication and multifunctionality, as well as perception aspects, such as sociocultural factors [12,25,26]. These findings also indicate that the indispensability of digital platforms is influenced by functional aspects and usage patterns, with variations in significance observed across countries. For example, platform comprehensiveness and security risks had a greater impact on the indispensability of the digital platform in Finland than in Korea, and social interaction capabilities had a greater impact in Korea than in Finland. 

This difference can be attributed to differences in the platform ecosystem and cultural characteristics of the two countries. Finland is dominated by global platforms such as Google, Apple, and Amazon. These global platforms frequently have concerns about security breaches. Therefore, Finns are forced to consider security risks when using these platforms. This can negatively affect perceptions of the indispensability of digital platforms. In Korea, on the other hand, market-leading local platforms such as Naver and Kakao have relatively low concerns about security breaches. Therefore, Koreans may not consider security risks as much when using these platforms. This can have a positive impact on perceptions of the indispensability of digital platforms. In addition, Koreans may be more dependent on social interaction through services provided in the home-grown local platforms. This may result in social interaction functions having a greater impact on the indispensability of digital platforms.

## 5. Conclusions

This study investigated the factors influencing the perceived indispensability of digital platforms across countries with varying gaps between global and local digital platforms by conducting surveys in Finland and Korea focused on the quality of digital platforms and their usage. The results reveal a higher perceived indispensability of digital platforms in Finland than in Korea. In both countries, usefulness and habitual platform use emerged as significant predictors of indispensability. However, the specific aspects of platform quality influencing this perception differed. In Finland, platform comprehensiveness and security risks were significantly and negatively associated with the perception of the indispensability of the digital platform, while social interaction features played a negligible role. In contrast, in Korea, social interaction was significantly and positively associated with the perception of the indispensability of the digital platform, while the effects of platform comprehensiveness and security risks were non-significant. These findings suggest that the indispensability of digital platforms is shaped by a combination of platform quality and usage patterns.

### 5.1. Implications for Researchers

For researchers, this research offers a deeper understanding of factors influencing the indispensability of the digital platform. This research identified specific aspects of platform quality influencing perceived indispensability (e.g., usefulness, security, comprehensiveness, and social interaction) as well as usage patterns (e.g., daily use frequency and habitual use). Future studies can further explore each aspect and the relationships among factors by examining how various aspects of platform quality interact with each other and usage patterns to shape indispensability. In addition, future studies can consider motivations for platform use by examining how intrinsic (e.g., enjoyment) and extrinsic (e.g., social pressure) motivations influence indispensability perception. In addition, this study found that the significance of the factors affecting indispensability vary significantly across countries, noting differences between Finland, where global platforms dominate, and Korea, where local platforms lead the market. This allows to expand the scope of digital platform research. By examining two distinct national contexts, this study extends the understanding of digital platform indispensability beyond that commonly studied the U.S. and Chinese markets, providing a more global perspective.

### 5.2. Implications for Policymakers

Given the platform gap between global and local platforms, this study offers some policy implications for Finland and Korea. This study highlights the importance of tailored regulatory approaches. In crafting policies, it is essential to consider the unique aspects of each country’s digital landscape, fostering a balance that supports local innovation while ensuring a competitive global presence. In Finland, where the findings indicate that platform comprehensiveness significantly and negatively impacts indispensability, the development of strong local platforms in each key area, such as online search, digital advertising, and mobile operating systems, is needed to reduce the country’s dependence on comprehensive global platforms. Policies supporting local development could strengthen the perception of indispensability by addressing specific gaps. Furthermore, policymakers, especially in Finland, should enforce stringent data privacy regulations to address user concerns about security risks. Ensuring robust legal frameworks for data protection can enhance user trust and platform indispensability. Second, Finland should consider regulatory measures to ensure fair competition between global and local platforms to level the playing field and prevent monopolistic practices. Regulatory measures aimed at ensuring fair competition may need to address security risks and comprehensiveness of platforms. Third, Finland should consider promoting digital literacy programs to help consumers make informed choices, understand the implications of relying on global platforms, and explore local alternatives. Policies promoting digital literacy align with the study’s findings that emphasize the importance of usefulness and habitual platform use. Educating users can enhance their understanding of these aspects, contributing to the perceived indispensability of platforms.

For Korea, our analysis shows that the nature of social interactions among users within domestic digital platforms has a significant impact on the indispensability of digital platforms in Korea. Therefore, to strengthen the indispensability of domestic platforms, it is necessary to develop policies that support the sustainability and competitiveness of domestic platforms such as Naver through incentives, subsidies, and regulations to protect domestic platforms. Second, Korea needs not only to support local platforms but also to encourage sound competition between global and local platforms to balance global and local competition while ensuring that domestic platforms have the opportunity to thrive without being overshadowed by global conglomerates. This includes providing incentives for innovation, funding for tech startups, and infrastructure support. Policies encouraging healthy competition while supporting domestic platforms align with the study’s results, emphasizing the significance of social interaction features. Balancing this competition can contribute to the perceived indispensability of local platforms. Third, Korea should consider incentives for research and development in the technology sector to help local platforms remain innovative and competitive on a global scale. Supporting research and development aligns with the study’s findings, emphasizing the importance of platform quality. Policies encouraging innovation can positively impact the perceived indispensability of digital platforms in Korea.

For both countries, this study highlights the usefulness and universal importance of habitual platform use. Both countries could benefit from policies promoting digital literacy. As the findings indicate that in Finland, a platform’s comprehensiveness and security safeguards are the most important factors for consumers, and that in Korea, the social interaction aspect of the platform is the most important, each country should consider these specific factors when devising relevant policies, which could reinforce the indispensable nature of the platform in each country.

### 5.3. Implications for Digital Platform Users

For digital platform users, this study offers information to make more informed decisions about digital platform choices by understanding the factors influencing the indispensability of the digital platform such as platform comprehensiveness, security risks, and social interaction features. Second, an understanding of the study’s findings will increase users’ digital literacy, enabling them to navigate and leverage platforms more effectively. This includes understanding the impact of habitual use and the importance of different platform qualities. Furthermore, understanding the factors influencing indispensability prompts users to engage with digital platforms more consciously. This can lead to a more intentional and satisfying digital experience. In addition, users may become more responsive to changes in platform features, recognizing their impact on indispensability. This adaptability allows users to navigate the evolving digital landscape with a greater understanding of their preferences. Third, users, especially those in diverse cultural contexts, can be more aware of the nuanced differences in views on platform indispensability. This study suggests that some user factors, such as usefulness and habitual use, offer insights into user behavior, making platform developers and marketers better meet user needs in different cultural contexts. This awareness can be crucial for individuals navigating digital spaces with varying platform ecosystems. Users may be encouraged to explore alternative platforms that align better with their preferences, particularly if the study highlights areas where local or global platforms excel or face challenges.

### 5.4. Implications for Practitioners

Platform developers in both Finland and Korea should prioritize the development of practical and efficient features that enhance user experiences. Emphasizing usability and functionality can increase the perceived indispensability of platforms. In addition, in Korea, developers should focus on integrating robust social interaction features to foster community building and engagement. These features can enhance user satisfaction and platform indispensability, particularly in collectivist cultures. Furthermore, given the significant negative impact of security concerns in Finland, developers should implement advanced security measures to protect user data and build trust. This includes regular security audits, transparent data protection policies, and user education on privacy practices.

For marketers, this study offers strategies that resonate with the unique cultural and technological contexts of each country. For example, emphasizing social connectivity and community features in Korea while highlighting security and practical functionality in Finland can improve user engagement and platform adoption.

### 5.5. Limitations

This study is not without limitations. First, this study focused on participants in Finland and Korea, thereby restricting the study’s subject. A more comprehensive interpretation could be achieved by extending the study to include other countries or considering other demographic factors. In addition, we recognize that our research design has inherent limitations, which we have sought to mitigate. One potential bias is the difference in sampling methods between the two countries. While the Finnish sample relied on convenience sampling, the Korean sample utilized a stratified random sampling method. To address this, we conducted robustness checks and controlled for demographic variables in our analysis to ensure that our findings were not unduly influenced by sampling differences. Future studies should aim to employ consistent methodologies for a more robust comparison. Second, this research specifically focused on Google and Naver—digital platforms that originated as search engines—among the numerous digital platforms available. Future research should broaden the scope, encompassing not only this category of digital platforms but also other types within the digital landscape. Last, despite this study adopting a cross-sectional approach to compare the two countries, the rapid development and proliferation of digital platforms suggest that an extended study duration could yield varied results. Therefore, it is imperative that future studies investigate how perceptions of the indispensability of digital platforms evolve using longitudinal data.

By building upon the findings of this study and acknowledging its limitations, future research can further refine the understanding of platform indispensability. This, in turn, can guide evidence-based policy interventions aimed at mitigating potential negative consequences and fostering a healthier digital ecosystem for all.

## Figures and Tables

**Table 1:** Respondent demographic information.

		Finland (N = 153)		Korea (N = 214)		M (SD)
Gender	Female	80	52%	142	66%	1.632 (0.510)
	Male	68	44%	72	34%	
	Other	5	3%	-	-	
Age	10s	3	2%	20	9%	3.376 (1.371)
	20s	43	28%	41	19%	
	30s	63	41%	46	21%	
	40s	23	15%	42	20%	
	50s	11	7%	44	21%	
	60s+	10	7%	21	10%	
Education	High school or less	17	11%	55	26%	2.771 (1.062)
	Some college	18	12%	24	11%	
	Bachelor’s degree	35	23%	116	54%	
	Graduate degree	83	54%	19	9%	
Monthly household income	<1500 USD	45	29%	32	15%	2.523 (1.042)
	1500–3000 USD	98	64%	69	32%	
	3000–4500 USD	8	5%	55	26%	
	4500–7000 USD	1	1%	38	18%	
	>7000 USD	1	1%	7	3%	

**Table 2:** Descriptive statistics (N = 367).

Variable	Min	Max	Mean	Standard Deviation
Indispensability	1	5	3.724	0.691
Comprehensiveness	1.75	5	3.993	0.559
Usefulness	2.25	5	4.033	0.516
Security risk	1	5	2.739	0.838
Social interaction	1.2	5	3.622	0.628
Habitual use	1.6	5	3.950	0.601
Daily use frequency	1	5	2.161	1.061

**Table 3:** Correlation analysis results.

	1	2	3	4	5	6	7	8	9	10	11
1. Indispensability	1										
2. Gender	0.05	1									
3. Age	−0.06	−0.03	1								
4. Education	0.08	−0.05	−0.08	1							
5. Income	0.05	−0.12 *	0.19 ^†^	0.10	1						
6. Comprehensiveness	0.36 ^‡^	0.09	−0.10	−0.09	−0.06	1					
7. Usefulness	0.59 ^‡^	0.10	−0.11 *	−0.03	0.01	0.54 ^‡^	1				
8. Security risk	−0.15 ^†^	0.01	−0.04	0.01	−0.13 *	−0.19	−0.20 ^†^	1			
9. Social interaction	0.44 ^‡^	−0.01	−0.16 ^†^	−0.03	−0.01	0.48 ^‡^	0.48 ^‡^	−0.05	1		
10. Habitual use	0.65 ^‡^	0.09	0.01	0.05	0.02	0.42 ^‡^	0.54 ^‡^	−0.15 ^†^	0.34 ^‡^	1	
11. Daily use frequency	0.32 ^‡^	−0.08	−0.15 ^†^	0.26 ^‡^	−0.05	0.07	0.22 ^‡^	−0.01	0.16 ^†^	0.31 ^‡^	1

Note: N = 367; * *p* < 0.05, ^†^ *p* < 0.01, ^‡^ *p* < 0.001.

**Table 4:** Country differences in digital platforms.

	Finland M (S.D.)	Korea M (S.D.)	Mean Difference
Indispensability	3.87 (0.53)	3.62 (0.77)	0.26 ***
Comprehensiveness	3.912 (0.50)	4.05 (0.59)	−0.13
Social interaction	3.70 (0.64)	3.57 (0.61)	0.14 *
Perceived usefulness	4.10 (0.41)	3.98 (0.58)	0.12 *
Perceived security risk	2.83 (0.95)	2.68 (0.75)	0.15
Habitual usage	4.04 (0.38)	3.89 (0.71)	0.16 *
Daily use frequency	2.51 (1.18)	1.91 (0.89)	0.60 ***

Note: using *t*-test; * *p* < 0.05, *** *p* < 0.001.

**Table 5:** Results of the hierarchical regression model (Finland).

Variable		Model 1		Model 2		Model 3	
		Coefficient	S.E.	Coefficient	S.E.	Coefficient	S.E.
Individual characteristics (Controls)	Gender	−0.010	0.079	−0.019	0.069	−0.014	0.066
	Age	−0.030	0.039	−0.005	0.034	0.006	0.033
	Education	0.051	0.044	0.055	0.039	0.044	0.038
	Income	0.051	0.054	0.045	0.047	0.063	0.045
Platform quality	Comprehensiveness			−0.131	0.087	−0.163 *	0.083
	Usefulness			0.535 ***	0.101	0.412 ***	0.101
	Security risk			−0.099 *	0.041	−0.094 *	0.039
	Social interaction			0.160 *	0.068	0.102	0.066
Platform usage	Habitual usage					0.400 ***	0.109
	Usage frequency					0.055	0.033
Constants		3.706 ***	0.267	1.653 **	0.539	0.680	0.573
R^2^		0.027		0.281		0.362	
Adjusted R^2^		0.000		0.241		0.318	
ΔF		1.011		7.027 ***		8.072 ***	
N		153		153		153	

Note: * *p* < 0.05, ** *p* < 0.01, *** *p* < 0.001; dependent variable: indispensability.

**Table 6:** Results of the hierarchical regression model (Korea).

Variable		Model 1		Model 2		Model 3	
		Coefficient	S.E.	Coefficient	S.E.	Coefficient	S.E.
Individual characteristics (Controls)	Gender	0.207	0.112	0.072	0.086	0.026	0.075
	Age	−0.016	0.036	0.043	0.029	0.015	0.025
	Education	−0.046	0.055	0.017	0.042	−0.018	0.037
	Income	0.084	0.048	0.040	0.038	0.030	0.033
Platform quality	Comprehensiveness			0.148	0.097	0.050	0.085
	Usefulness			0.565 ***	0.106	0.327 ***	0.096
	Security risk			0.020	0.057	0.041	0.050
	Social interaction			0.290 ***	0.085	0.199 **	0.074
Platform usage	Habitual usage					0.477 ***	0.065
	Usage frequency					0.067	0.043
Constants		3.706 ***	0.267	1.653 **	0.539	0.680	0.573
R^2^		0.029		0.452		0.592	
Adjusted R^2^		0.010		0.430		0.572	
ΔF		1.557		21.096 ***		29.461 ***	
N		214		214		214	

Note: ** *p* < 0.01, *** *p* < 0.001; dependent variable: indispensability.

**Table 7:** Analysis results of hierarchical regression, Model 3.

Variable		Finland		Korea	
		Coefficient	S.E.	Coefficient	S.E.
Individual characteristics (Controls)	Gender	−0.01	0.07	0.03	0.08
	Age	0.01	0.03	0.02	0.03
	Education	0.04	0.04	−0.02	0.04
	Income	0.06	0.05	0.03	0.03
Platform quality	Comprehensiveness	−0.16 *	0.08	0.05	0.09
	Usefulness	0.41 ***	0.10	0.33 ***	0.10
	Security risk	−0.09 *	0.04	0.04	0.05
	Social interaction	0.10	0.07	0.20 **	0.07
Platform usage	Habitual usage	0.40 ***	0.11	0.48 ***	0.07
	Usage frequency	0.06	0.03	0.07	0.04
Constants		3.706 ***	0.267	1.653 **	0.539
R^2^		0.362		0.592	
Adjusted R^2^		0.318		0.572	
N		153		214	

Note: * *p* < 0.05, ** *p* < 0.01, *** *p* < 0.001; dependent variable: indispensability.

## Data Availability

The data are available from the corresponding author upon reasonable request.

## References

[1] Van Dijck J., Poell T., De Waal M. (2018). The Platform Society: Public Values in a Connective World.

[2] Quarta A. (2020). Narratives of the Digital Economy: How Platforms Are Challenging Consumer Law and Hierarchical Organization. Glob. Jurist.

[3] Vaidhyanathan S. (2018). Antisocial Media: How Facebook Disconnects Us and Undermines Democracy.

[4] Pierson J. (2021). Digital Platforms as Entangled Infrastructures: Addressing Public Values and Trust in Messaging Apps. Eur. J. Commun..

[5] Hoffman D.L., Novak T.P., Venkatesh A. (2004). Has the Internet Become Indispensable?. Commun. ACM.

[6] Hermes S., Clemons E., Schreieck M., Pfab S., Mitre M., Böhm M., Wiesche M., Krcmar H. (2020). Breeding Grounds of Digital Platforms: Exploring the Sources of American Platform Domination, China’s Platform Self-Sufficiency, and Europe’s Platform Gap. Res. Pap..

[7] Chiarella M.L. (2023). Digital Markets Act (DMA) and Digital Services Act (DSA): New Rules for the EU Digital Environment. Athens JL.

[8] Beems B. (2023). The DMA in the Broader Regulatory Landscape of the EU: An Institutional Perspective. Eur. Compet. J..

[9] EU Artificial Intelligence Act 2024. https://artificialintelligenceact.eu/.

[10] Neuwirth R.J. (2023). Prohibited Artificial Intelligence Practices in the Proposed EU Artificial Intelligence Act (AIA). Comput. Law Secur. Rev..

[11] McKnight S., Kenney M., Breznitz D. (2023). Regulating the platform giants: Building and governing China's online economy. Policy Internet.

[12] Zhang Z. (2021). Infrastructuralization of Tik Tok: Transformation, Power Relationships, and Platformization of Video Entertainment in China. Media Cult. Soc..

[13] Kim K.P. (2023). The Rise and Development of the Platform Economy in South Korea. Int. J. Asian Stud..

[14] Yoon J.H. “NAVER Shaken by Google’ Advance?” Money Today 2023. https://news.mt.co.kr/mtview.php?no=2023061213434572239.

[15] Anttiroiko A.V. (2016). City-as-a-Platform: The Rise of Participatory Innovation Platforms in Finnish Cities. Sustainability.

[16] Platzer E., Petrovic O., Rauch W., Maxl E. (2009). An Experimental Deprivation Study on Technology Indispensability. Central European Conference on Information and Intelligent Systems.

[17] Rahman K.S. (2018). Regulating Informational Infrastructure: Internet Platforms as the New Public Utilities. Georget. Law Technol. Rev..

[18] Marinelli A., Parisi S. (2024). Apps, Platforms, and Everyday Practices: How People Perceive and Care (or not) About the Digital Traces They Leave Online. Am. Behav. Sci..

[19] Schnauber-Stockmann A., Mangold F. (2020). Day-to-day routines of media platform use in the digital age: A structuration perspective. Commun. Monogr..

[20] Barns S. (2019). Negotiating the platform pivot: From participatory digital ecosystems to infrastructures of everyday life. Geogr. Compass.

[21] Fast K. (2018). A Discursive Approach to Mediatisation: Corporate Technology Discourse and the Trope of Media Indispensability. Media Commun..

[22] Hoffman D.L. (2012). Internet Indispensability, Online Social Capital, and Consumer Well-Being. Transformative Consumer Research for Personal and Collective Well-Being.

[23] Cheng Z., Cheng Z., Cheng Z. On Smartphone Indispensability: A Country-level Exploratory Study. Proceedings of the AMCIS 2015.

[24] Kinder E., Jarrahi M.H., Sutherland W. (2019). Gig platforms, tensions, alliances and ecosystems: An actor-network perspective. Proc. ACM Hum.-Comput. Interact..

[25] Jansson A. (2015). The Molding of Mediatization: The Stratified Indispensability of Media in Close Relationships. Communications.

[26] Plantin J.C., De Seta G. (2019). WeChat as Infrastructure: The Techno-Nationalist Shaping of Chinese Digital Platforms. Chin. J. Commun..

[27] He Q. (2022). Rethinking the Legal Regulation of Internet Platform Monopoly in China. Policy Internet.

[28] Chirita A. (2021). Abuse of Global Platform Dominance or Competition on the Merits?. Loy. Consum. Lit. Rev..

[29] Barwise P., Watkins L. (2018). The evolution of digital dominance. Digital Dominance: The Power of Google, Amazon, Facebook, and Apple.

[30] Marty F., Mouton J. (2022). Ecosystems as Quasi-Essential Facilities: Should We Impose Platform Neutrality?. J. Law Mark. Innov..

[31] Moore M., Tambini D. (2022). Regulating Big Tech: Policy Responses to Digital Dominance.

[32] Flew T., Gillett R. (2021). Platform policy: Evaluating different responses to the challenges of platform power. J. Digit. Media Policy.

[33] Cammaerts B., Mansell R. (2020). Digital platform policy and regulation: Toward a radical democratic turn. Int. J. Commun..

[34] Nadeem S. (2024). Platform Power: Investigating the Dynamics of Dominant Platforms and their Impact on Industry Competition. Res. Stud. Bus..

[35] Qiu J.L., Curtin M., Shah H. (2010). Chinese Techno-Nationalism and Global wi-fi Policy. Reorienting Global Communication: Indian and Chinese Media Beyond Borders.

[36] Bouwman H., Carlsson C., Carlsson J., Nikou S., Sell A., Walden P. How Nokia Failed to Nail the Smartphone Market. Proceedings of the 25th European Regional Conference of the International Telecommunications Society (ITS): “Disruptive Innovation in the ICT Industries: Challenges for European Policy and Business”.

[37] Hofstede G. (2016). Culture’s consequences: Comparing values, behaviors, institutions, and organizations across nations. Coll. Aviat. Rev..

[38] Straub D., Keil M., Brenner W. (1997). Testing the technology acceptance model across cultures: A three country study. Inf. Manag..

[39] McIntyre D.P., Srinivasan A. (2017). Networks, Platforms, and Strategy: Emerging Views and Next Steps. Strateg. Manag. J..

[40] Claussen J., Kretschmer T., Mayrhofer P. (2013). The effects of rewarding user engagement: The case of Facebook apps. Inf. Syst. Res..

[41] Asante I.O., Jiang Y., Hossin A.M., Luo X. (2023). Optimization of consumer engagement with artificial intelligence elements on electronic commerce platforms. J. Electron. Commer. Res..

[42] Cheng S., Lee S.-J., Choi B. (2019). An Empirical Investigation of Users’ Voluntary Switching Intention for Mobile Personal Cloud Storage Services based on the Push-Pull-Mooring Framework. Comput. Hum. Behav..

[43] Davis F.D., Granić A., Marangunić N. (2023). The technology acceptance model 30 years of TAM. Technology.

[44] Baker-Eveleth L., Stone R.W. (2020). User’s perceptions of perceived usefulness, satisfaction, and intentions of mobile application. Int. J. Mob. Commun..

[45] Gefen D., Karahanna E., Straub D.W. (2003). Trust and TAM in online shopping: An integrated model. MIS Q..

[46] Thies F., Wessel M., Benlian A. (2016). Effects of social interaction dynamics on platforms. J. Manag. Inf. Syst..

[47] Vakeel K.A., Malthouse E.C., Yang A. (2021). Impact of network effects on service provider performance in digital business platforms. J. Serv. Manag..

[48] Lee M.C. (2009). Factors Influencing the Adoption of Internet Banking: An Integration of TAM and TPB with Perceived Risk and Perceived Benefit. Electron. Commer. Res. Appl..

[49] Quach S., Thaichon P., Martin K.D., Weaven S., Palmatier R.W. (2022). Digital technologies: Tensions in privacy and data. J. Acad. Mark. Sci..

[50] Isaak J., Hanna M.J. (2018). User Data Privacy: Facebook, Cambridge Analytica, and Privacy Protection. Computer.

[51] Duan Y., Ge Y., Feng Y. (2022). Pricing and personal data collection strategies of online platforms in the face of privacy concerns. Electron. Commer. Res..

[52] Anderson I.A., Wood W. (2021). Habits and the Electronic Herd: The Psychology Behind Social Media’s Successes and Failures. Consum. Psychol. Rev..

[53] Bjur J., Schrøder K.C., Hasebrink U., Courtois C., Adoni H., Nossek H. (2013). Cross-media use: Unfolding complexities in contemporary audiencehood. Audience Transformations.

[54] Büchi M., Just N., Latzer M. (2016). Modeling the second-level digital divide: A five-country study of social differences in Internet use. New Media Soc..

[55] Liao C., Palvia P., Lin H.N. (2006). The Roles of Habit and Web Site Quality in E-Commerce. Int. J. Inf. Manag..

[56] Aydin M. (2022). A Multilevel Modeling Approach to Investigating Factors Impacting Computer and Information Literacy: ICILS Korea and Finland Sample. Educ. Inf. Technol..

[57] Jang M., Aavakare M., Nikou S., Kim S. (2021). The Impact of Literacy on Intention to Use Digital Technology for Learning: A Comparative Study of Korea and Finland. Telecommun. Policy.

[58] OECD Data Usage Increases More than 25% in the Majority of OECD Countries in 2019. www.oecd.org/digital/broadband-statistics-update.

[59] Plantin J.-C., Lagoze C., Edwards P.N., Sandvig C. (2018). Infrastructure Studies Meet Platform Studies in the Age of Google and Facebook. New Media Soc..

[60] Cothran T. (2011). Google Scholar Acceptance and Use Among Graduate Students: A Quantitative Study. Libr. Inf. Sci. Res..

[61] Limayem M., Hirt S.G., Cheung C.M. (2007). How Habit Limits the Predictive Power Of Intention: The Case of Information Systems Continuance. MIS Q..

[62] Matute J., Polo-Redondo Y., Utrillas A. (2016). The Influence of EWOM Characteristics on Online Repurchase Intention: Mediating Roles of Trust and Perceived Usefulness. Online Inf. Rev..

[63] Forsythe S., Liu C., Shannon D., Gardner L.C. (2006). Development of a Scale to Measure the Perceived Benefits and Risks of Online Shopping. J. Interact. Mark..

[64] Gong Z., Han Z., Li X., Yu C., Reinhardt J.D. (2019). Factors Influencing the Adoption of Online Health Consultation Services: The Role of Subjective Norm, Trust, Perceived Benefit, and Offline Habit. Front. Public Health.

[65] Lu Y., Cao Y., Wang B., Yang S. (2011). A Study on Factors that Affect Users’ Behavioral Intention to Transfer Usage from the Offline to the Online Channel. Comput. Hum. Behav..

[66] Ridings C.M., Gefen D. (2004). Virtual Community Attraction: Why People Hang Out Online. J. Comput.-Mediat. Commun..

[67] Seol S., Lee H., Yu J., Zo H. (2016). Continuance Usage of Corporate SNS Pages: A Communicative Ecology Perspective. Inf. Manag..

[68] Lee W.K. (2014). The Temporal Relationships Among Habit, Intention and IS Uses. Comput. Hum. Behav..

[69] Hubert M., Blut M., Brock C., Backhaus C., Eberhardt T. (2017). Acceptance of Smartphone-Based Mobile Shopping: Mobile Benefits, Customer Characteristics, Perceived Risks, and the Impact of Application Context. Psychol. Mark..

[70] Fornell C., Larcker D.F. (1981). Evaluating Structural Equation Models with Unobservable Variables and Measurement Error. J. Mark. Res..

[71] Musil C.M., Jones S.L., Warner C.D. (1998). Structural Equation Modeling and Its Relationship to Multiple Regression and Factor Analysis. Res. Nurs. Health.

[72] Nusair K., Hua N. (2010). Comparative Assessment of Structural Equation Modeling and Multiple Regression Research Methodologies: E-commerce Context. Tour. Manag..

[73] Petrocelli J.V. (2003). Hierarchical Multiple Regression in Counseling Research: Common Problems and Possible Remedies. Meas. Eval. Couns. Dev..

[74] Falvo M.J., Earhart G.M. (2009). Six-minute Walk Distance in Persons with Parkinson Disease: A Hierarchical Regression Model. Arch. Phys. Med. Rehabil..

[75] Yang F. (2022). The postcolonial route of WeChat: Technological mimicry, excess, and orientalism. Asian J. Commun..

[76] Wörsdörfer M. (2023). The Digital Markets Act and EU competition policy: A critical ordoliberal evaluation. Philos. Manag..

[77] Hwang I.J., Lee B.G., Kim K.Y. (2014). Information asymmetry, social networking site word of mouth, and mobility effects on social commerce in Korea. Cyberpsychology Behav. Soc. Netw..

[78] Kim J., Kim S., Beck S., Jung K. Why Google cannot be the # 1 in Korea? In Search for Critical Success Factors from Local User Experience. Proceedings of the 33rd Annual ACM Conference Extended Abstracts on Human Factors in Computing Systems.

